# *Vibrio cholerae* O1 associated with recent endemic cholera shows temporal changes in serotype, genotype, and drug-resistance patterns in Bangladesh

**DOI:** 10.1186/s13099-023-00537-0

**Published:** 2023-04-12

**Authors:** Fatema Tuz Jubyda, Kazi Sumaita Nahar, Indrajeet Barman, Fatema-Tuz Johura, Mohammad Tarequl Islam, Marzia Sultana, Wali Ullah, Jarin Tasnim, Sahitya Ranjan Biswas, Md Mamun Monir, Christine Marie George, Andrew Camilli, Niyaz Ahmed, Allen G. Ross, John D. Clemens, Munirul Alam

**Affiliations:** 1 icddr,b (International Centre for Diarrhoeal Disease Research, Bangladesh), 68, Shaheed Tajuddin Ahmed Sharani, Mohakhali, Dhaka, 1212 Bangladesh; 2 Department of Microbiology, Jahangirnagar University, Dhaka, Bangladesh; 3 Johns Hopkins Bloomberg School of Public Health, Baltimore, MD USA; 4 Tufts University, Boston, MA USA; 5 Pathogen Biology Laboratory, Department of Biotechnology and Bioinformatics, University of Hyderabad, Hyderabad, Telangana State India; 6 Charles Sturt University, Orange, NSW Australia

**Keywords:** *Vibrio cholerae*, Temporal transition, Multi-drug resistant

## Abstract

**Background:**

Despite the advancement in our understanding of cholera and its etiological agent, *Vibrio cholerae*, the prevention and treatment of the disease are often hindered due to rapid changes in drug response pattern, serotype, and the major genomic islands namely, the CTX-prophage, and related genetic characteristics*.* In the present study, *V. cholerae* (n = 172) associated with endemic cholera in Dhaka during the years 2015–2021 were analyzed for major phenotypic and genetic characteristics, including drug resistance patterns.

**Results:**

Results revealed that the *V. cholerae* strains belonged to serogroup O1 biotype El Tor carrying El Tor -specific genes *rtxC*, *tcpA* El Tor, and *hlyA* El Tor, but possessed classical-biotype cholera toxin. Serotypes of *V. cholerae* strains differed temporally in predominance with Inaba during 2015–2017, and again in 2020–2021, while Ogawa was the predominant serotype in 2018–2019. Also, *ctxB1* was predominant in *V. cholerae* associated with cholera during 2015–2017, while *ctxB7* was predominant in 2018, and in the subsequent years, as observed until 2021. *V. cholerae* strains differed in their antibiotic resistance pattern with a majority (97%) being multi-drug resistant (MDR) and belonging to six sub-groups. Notably, one of these MDR strains was resistant to eleven of the eighteen antibiotics tested, with resistance to fourth-generation cephalosporin (cefepime), and aztreonam. This extreme drug resistant (XDR) strain carried resistance-related genes namely, extended-spectrum *β*-lactamases (ESBL), *blaOXA*-1 and *blaPER*-3.

**Conclusion:**

The observed temporal switching of serotypes, as well as the *ctxB* genotype, and the emergence of MDR/XDR *V. cholerae* and their association with endemic cholera in Dhaka underscore the need for routine monitoring of the pathogen for proper patient management.

## Background

Cholera is an infectious diarrheal disease, which if not treated timely, can be fatal [1]. The disease has been endemic in the Ganges Delta of the Bay of Bengal, Bangladesh and India for centuries [2]. The etiological agent of the disease is a Gram negative Gammaproteobacteria, *Vibrio cholerae*. Based on the variation of ‘O’ antigenic lipopolysaccharide (LPS), more than 200 serogroups of *V. cholerae* have been identified, of which serogroups O1 and O139 are primarily associated with epidemic cholera [3]. *V. cholerae* O1 has two biotypes, classical and El Tor. The two biotypes are universally recognized, differing in major phenotypic and genetic characteristics [4], including genes associated with important virulence factors, for example, toxin coregulated pilus (*tcpA*) and cholera toxin B-subunit (*ctxB*) [5]. Seven pandemics of cholera have been documented, with the seventh pandemic being the longest that started in the 1960s and is still present. Of the seven recognized cholera pandemics, the first six are believed to have been caused by the classical biotype of *V. cholerae* O1, and the ongoing seventh pandemic is being caused by the El Tor biotype.

The seventh cholera pandemic is significantly different from the others partly because of its rapid global spread and remarkable evolution of *V. cholerae*. The emergence of atypical El Tor variants with a few classical biotype attributes and its global spread, the predominance of Haitian variants in endemic regions of Asia and Africa, and the acquisition of virulence-related gene islands are examples of a few documented evolutionary events that potentially changed the course of the disease scenario [2, 4, 6–9]. Recent evolutionary genetic changes in *V. cholerae* O1 El Tor include several non-synonymous mutations in the cholera toxin B (*ctxB*) gene, resulting in alleles such as *ctxB*1 and *ctxB*7 [9, 10]. With documentation of the emerging new alleles, there are records of notable events such as periodic switching of serotype and *ctxB* genotype. For instance, following the spread of the Haitian variant *ctxB* allele, there was evidence of switching of the classical and Haitian *ctxB* in endemic regions [11]. As far we know, there is no evidence of an association between these two genotypes and the periodic switching with cholera outbreak in Bangladesh. Similarly, serotype conversion from Inaba to Ogawa, and back to Inaba again was also documented for many regions [11, 12]. There have been a few speculations to describe this periodic serotype switching, which presumably is attributable to the selection pressures prevailing in the environments [11]. Although the epidemiological implications of these switching are yet to be understood, these events are presumed to have a definite genetic basis and could therefore contribute to the infectious propensity of *V. cholerae* O1 [12].

Cholera intervention includes treatment of the affected individuals with an effective antibiotic alongside the oral rehydration therapy, although *V. cholerae* has already developed resistance towards a number of antimicrobials posing new challenges in the clinical management of cholera. Such antibiotic resistance is associated with the acquisition of mobile genetic elements such as the SXT integrative and conjugative element (ICE) by *V. cholerae*. The SXT-ICE contributes not only to the spread of antimicrobial resistance genes, but also drives *V. cholerae* diversity [13]. In the present study, *V. cholerae* O1 associated with endemic cholera in Dhaka during 2015–2021, were analyzed for major phenotypic (culture, serology, and antimicrobial susceptibility assay) and genetic characteristics, including the drug response pattern. The results presented in this study highlight a few notable temporal changes in the genotypic and phenotypic characteristics, including the emergence of an extreme drug-resistant *V. cholerae* O1 strain, providing new insights that could aid the prevention of cholera in Bangladesh.

## Methods

### Bacterial strains

In this study, a set of 172 *Vibrio cholerae* O1 strains were isolated from clinical samples collected in Dhaka, Bangladesh, between 2015 and 2021, as a part of an epidemiological surveillance conducted by the International Centre for Diarrhoeal Disease Research, Bangladesh (icddr,b). The strains were isolated from the stool of suspected cholera patients seeking treatment at icddr,b Dhaka Hospital. Informed consent was obtained from the patients or legal guardians for minor patients prior to the collection of stool samples. For the years 2015, 2016, and 2017, we tried to select strains covering each month of the year, given that *V. cholerae* O1 was isolated in those months. For the years 2018–2020, the rate of isolation of *V. cholerae* O1 decreased drastically. Therefore, we selected almost all the strains that were isolated in those years. *V. cholerae* O1 isolation rate increased again in 2021 and we selected strains representing each month until the studied period. Isolation and identification of *V. cholerae* were performed according to standard cultural and molecular methods as described previously [14]. Genomic DNA was extracted from the strains following boiling lysis method, described elsewhere [5]. Additionally, genomic DNA of the XDR strain was extracted using Qiagen Blood and Tissue Kit as per the manufacturer’s instructions.

### Serogrouping

The serogroup of the *V. cholerae* isolates was confirmed by slide agglutination test, using *V. cholerae* O1 and O139 specific polyvalent antisera and were tested further using serotype specific monoclonal antibodies, Inaba and Ogawa as described previously [5]. For molecular confirmation of serogroups, and detection of cholera toxin, multiplex PCR targeting O1-(*wbe*), O139-(*wbf*), and *ctxA* genes were performed [10].

### Antibiotic susceptibility assay

Bacterial susceptibility to antimicrobials was determined by standard disc diffusion test on Muller-Hinton agar (BD, USA) according to Clinical and Laboratory Standards Institute guidelines as described previously [5]. All strains of *V. cholerae* were tested for susceptibility to ampicillin (AMP, 10 μg), ceftriaxone (CRO, 30 μg), ciprofloxacin (CIP, 5 μg), mecillinam (MEL, 25 μg), erythromycin (E, 15 μg), nalidixic acid (NA, 30 μg), imipenem (IMP, 10 μg), sulfamethoxazole/trimethoprim (SXT, 25 μg), streptomycin (S, 10 μg), levofloxacin (LEV, 5 μg), cephalothin (KF, 30 μg), cefixime (CFM, 5 μg), cefepime (FEP, 30 μg), tetracycline (TE, 30 μg), aztreonam (ATM, 30 μg), azithromycin (AZM, 15 μg), chloramphenicol (C, 30 μg), and gentamicin (CN, 10 μg) using commercially available discs (BD BBL SensiDisc). The minimum inhibitory concentration (MIC) was determined by E-test (Biomeuriex). Suspected Extended Spectrum Beta Lactamase (ESBL) producing *V. cholerae* isolates were screened for the production of ESBLs by using a double disk synergy test (DDST) [15].

### Polymerase chain reaction (PCR) assay

PCR assays were performed to detect genes encoding zonula occludens toxin (*zot*), accessory cholera enterotoxin (*ace*), hemolysin (*hlyA*), SXT-related integrase (*intSXT*) and biotype-specific (El Tor and Classical) phage encoded repressor (*rstR*), toxin coregulated pilus (*tcpA*), and repeat in toxin (*rtxC*) using primers and conditions described previously [5]. All antibiotic-resistant *V. cholerae* O1 strains were tested for gene encoding the class 1 and class 2 integron using primers and procedures described elsewhere [16]. Double mismatch amplification mutation assay (DMAMA)-PCR was performed to determine three genotypes of the cholera toxin gene, Classical (*ctxB* genotype 1, *ctxB1*), El Tor (*ctxB* genotype 3, *ctxB3*), and Haitian type (*ctxB* genotype 7, *ctxB7*) based on nucleotide substitution at position 58, 115, and 203 [17]. *V. cholerae* O1 strains O395 (Classical), N16961 (El Tor), and EL-1786 (Haitian variant, *ctxB*7) were used as control for the PCR.

PCR was also performed to detect genes for ESBL, AmpC, carbapenemase, and quinolone resistance [18–22]. Specifically, multiplex PCR (6 sets) was performed to detect ESBL genes *blaTEM/blaSHV/blaOXA*-1-like genes, *blaCTX-M* including phylogenetic groups 1, 2 and 9, carbapenemase genes *blaVEB/blaGES/blaPER, blaVIM/blaIMP/blaKPC, blaGES/blaOXA-*48- like genes, and the AmpC group of genes *blaMOX/blaFOX/blaEBC/blaCIT/blaDHA/blaACC.* Simplex PCR was used to detect the following genes: ESBL (*blaCTX-M*-8/-25) [18]; carbapenemase (*blaAIM, blaGIM, blaSIM, and blaDIM*) [19]; *blaNDM* [22], quinolone resistance related *qnrVC* [20], efflux pump encoding *qepA*, acetylase encoding *aac(6’)-Ib-cr* [21].

### Sequencing of antibiotic resistant genes

PCR amplified antibiotic resistant genes were sequenced using an ABI Big-Dye Terminator v.3.1 Cycle Sequencing Reaction kit (Applied Biosystems) on an ABI PRISM 3500 XL genetic analyzer (AppliedBiosystems, Foster City, USA). The BLASTN program (www.ncbi.nlm.nih.gov/BLAST) was used for homology searching. The partial sequences of the genes were submitted to GenBank (Accession Numbers: MK992813, MK992814, MK992815).

### MLST and MLVA typing

We examined the draft genome assemblies of the 30 recently sequenced strains from 2018 to 2019 [23] and determined their multi-locus sequence type (MLST) based on seven housekeeping genes, *adk, gyrB, mdh, metE, pntA, purM, and pyrC*, using PubMLST scheme [24]. Additionally, multiple-locus variable-number tandem repeat (MLVA) analysis of selected isolates were performed based on five MLVA loci (VC01747, VC0436, VC1650, VCA0171 & VCA0283) following methods as described previously [25].

## Results

### Phenotypic and genotypic characteristics

**Fig. 1:**
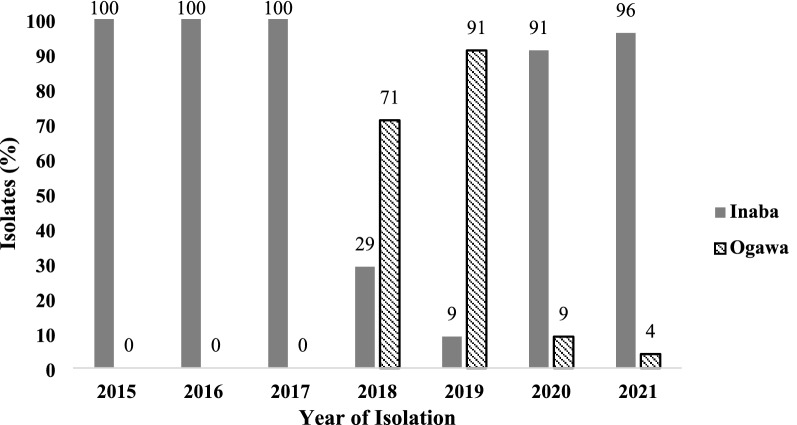
Temporal distribution of serotypes among *Vibrio cholerae* O1 strains isolated from Dhaka, 2015–2021

All of the total 172 isolates tested produced characteristic colonies typical of *V. cholerae* on both thiosulfate citrate bile-salts sucrose (TCBS) agar and taurocholate tellurite gelatin agar (TTGA). Positive agglutination with a polyvalent antibody for *V. cholerae* serogroup O1 followed by positive agglutination with monovalent Inaba (143/172) and Ogawa antisera (29/172) confirmed both Inaba and Ogawa serotypes were associated with endemic cholera in Dhaka, Bangladesh (Table 1). Although, the serotypes of *V. cholerae* strains differed temporally in predominance, one or the other of the two serotypes dominated in clinical cholera cases in a given year. For instance, Inaba was exclusively found to be associated with cholera in the first 3 years (2015–2017) while Ogawa was not found among the strains tested during this period. Thereafter, Ogawa was found to be the predominant serotype (71–91%) in 2018–2019, which then dipped to a low (9–4%) in 2020–2021 with the sharp rise of the Inaba serotype strains (91–96%) (Fig. 1). Overall, Inaba was the predominant serotype among the *V. cholerae* O1 strains associated with cholera for most of the study period.

**Table 1:** Genetic characteristics and drug resistance profile of *Vibrio cholerae* O1 strains isolated from Dhaka, 2015–2021

Year of isolation	No. of strains	Serotype	wbeO1	ace	zot	tcpA	rtxC	ctxA	ctxB type	rstR	hlyA	intSXT	intl1	Drug resistance profile	Deduced genetic characteristics and AMR profile	MLST type	MLVA type
2015	16	Inaba	+	+	+	ET	+	+	B1	ET	ET	+	–	NA, S, SXT	wbeO1 + , ace + , zot + , tcpA(ET), rtxC + , ctxA + , rstR(ET), hlyA(ET), Inaba, ctxB1, intSXT + , intl1–, NA, S, SXT	ND	ND
	1	Inaba	+	+	+	ET	+	+	B7	ET	ET	+	–	NA, S, SXT	wbeO1 + , ace + , zot + , tcpA(ET), rtxC + , ctxA + , rstR(ET), hlyA(ET), Inaba, ctxB7, intSXT + , intl1–, NA, S, SXT	ND	ND
	3	Inaba	+	+	+	ET	+	+	B1	ET	ET	–	–	NA	wbeO1 + , ace + , zot + , tcpA(ET), rtxC + , ctxA + , rstR(ET), hlyA(ET), Inaba, ctxB1, intSXT–, intl1–,NA	ND	ND
2016	29	Inaba	+	+	+	ET	+	+	B1	ET	ET	+	–	NA, S, SXT	wbeO1 + , ace + , zot + , tcpA(ET), rtxC + , ctxA + , rstR(ET), hlyA(ET), Inaba, ctxB1, intSXT + , intl1–, NA, S, SXT	ND	ND
	1	Inaba	+	+	+	ET	+	+	B7	ET	ET	+	–	NA, S, SXT	wbeO1 + , ace + , zot + , tcpA(ET), rtxC + , ctxA + , rstR(ET), hlyA(ET), Inaba, ctxB7, intSXT + , intl1–, NA, S, SXT	ND	ND
	1	Inaba	+	+	+	ET	+	+	B1	ET	ET	–	–	NA	wbeO1 + , ace + , zot + , tcpA(ET), rtxC + , ctxA + , rstR(ET), hlyA(ET), Inaba, ctxB1, intSXT–, intl1–, NA	ND	ND
2017	32	Inaba	+	+	+	ET	+	+	B1	ET	ET	+	–	NA, S, SXT	wbeO1 + , ace + , zot + , tcpA(ET), rtxC + , ctxA + , rstR(ET), hlyA(ET), Inaba, ctxB1, intSXT + , intl1–, NA, S, SXT	ND	ND
2018	2	Inaba	+	+	+	ET	+	+	B1	ET	ET	+	–	NA, SXT	wbeO1 + , ace + , zot + , tcpA(ET), rtxC + , ctxA + , rstR(ET), hlyA(ET), Inaba, ctxB1, intSXT + , intl1–, NA, SXT	ST69	1
	1	Inaba	+	+	+	ET	+	+	B1	ET	ET	+	–	NA, SXT		ST69	4
	1	Inaba	+	+	+	ET	+	+	B1	ET	ET	+	–	NA, S, SXT	wbeO1 + , ace + , zot + , tcpA(ET), rtxC + , ctxA + , rstR(ET), hlyA(ET), Inaba, ctxB1, intSXT + , intl1–, NA, S, SXT	ST69	1
	1	Inaba	+	+	+	ET	+	+	B1	ET	ET	+	–	NA, S, SXT		ST69	2
	1	Ogawa	+	+	+	ET	+	+	B7	ET	ET	+	–	NA	wbeO1 + , ace + , zot + , tcpA(ET), rtxC + , ctxA + , rstR(ET), hlyA(ET), Ogawa, ctxB7, intSXT + , intl1–, NA	ST69	5
	3	Ogawa	+	+	+	ET	+	+	B7	ET	ET	+	–	NA, S, SXT	wbeO1 + , ace + , zot + , tcpA(ET), rtxC + , ctxA + , rstR(ET), hlyA(ET), Ogawa, ctxB7, intSXT + , intl1–, NA, S, SXT	ST69	3
	4	Ogawa	+	+	+	ET	+	+	B7	ET	ET	+	–	NA, S, SXT		ST69	5
	1	Inaba	+	+	+	ET	+	+	B7	ET	ET	+	+	NA, S, SXT	wbeO1 + , ace + , zot + , tcpA(ET), rtxC + , ctxA + , rstR(ET), hlyA(ET), Inaba, ctxB7, intSXT + , intl 1 + , NA, S, SXT	ND	ND
	3	Ogawa	+	+	+	ET	+	+	B7	ET	ET	+	–	NA, S, SXT, MEL	wbeO1 + , ace + , zot + , tcpA(ET), rtxC + , ctxA + , rstR(ET), hlyA(ET), Ogawa, ctxB7, intSXT + , intl1–, NA, S, SXT, MEL	ST69	3
	2	Ogawa	+	+	+	ET	+	+	B7	ET	ET	+	–	NA, S, SXT, MEL		ST69	5
	1	Ogawa	+	+	+	ET	+	+	B7	ET	ET	+	+	NA, S, SXT, MEL	wbeO1 + , ace + , zot + , tcpA(ET), rtxC + , ctxA + , rstR(ET), hlyA(ET), Ogawa, ctxB7, intSXT + , intl1 + , NA, S, SXT, MEL	ST69	5
	1	Ogawa	+	+	+	ET	+	+	B7	ET	ET	+	+	NA, S, SXT	wbeO1 + , ace + , zot + , tcpA(ET), rtxC + , ctxA + , rstR(ET), hlyA(ET), Ogawa, ctxB7, intSXT + , intl1 + , NA, S, SXT	ST69	6
2019	5	Ogawa	+	+	+	ET	+	+	B7	ET	ET	+	–	NA, S, SXT	wbeO1 + , ace + , zot + , tcpA(ET), rtxC + , ctxA + , rstR(ET), hlyA(ET), Ogawa, ctxB7, intSXT + , intl1–, NA, S, SXT	ST69	5
	3	Ogawa	+	+	+	ET	+	+	B7	ET	ET	+	–	NA, S, SXT		ST69	3
	1	Ogawa	+	+	+	ET	+	+	B7	ET	ET	+	+	NA, S, SXT	wbeO1 + , ace + , zot + , tcpA(ET), rtxC + , ctxA + , rstR(ET), hlyA(ET), Ogawa, ctxB7, intSXT + , intl1 + , NA, S, SXT	ST69	5
	1	Ogawa	+	+	+	ET	+	+	B7	ET	ET	+	+	NA, S, SXT	wbeO1 + , ace + , zot + , tcpA(ET), rtxC + , ctxA + , rstR(ET), hlyA(ET), Ogawa, ctxB7, intSXT + , intl1 + , NA, S, SXT	ND	ND
	1	Inaba	+	+	+	ET	+	+	B1	ET	ET	+	–	KF, S, CFM, CRO, NA, SXT, FEP, MEL, CIP, AMP, ATM	wbeO1 + , ace + , zot + , tcpA(ET), rtxC + , ctxA + , rstR(ET), hlyA(ET), Inaba, ctxB1, KF, S, CFM, CRO, NA, SXT, FEP, MEL, CIP, AMP, ATM	ST69	2
2020	31	Inaba	+	+	+	ET	+	+	B7	ET	ET	+	–	NA, S, SXT	wbeO1 + , ace + , zot + , tcpA(ET), rtxC + , ctxA + , rstR(ET), hlyA(ET), Inaba, ctxB7, intSXT + , intl1–, NA, S, SXT	ND	ND
	3	Ogawa	+	+	+	ET	+	+	B7	ET	ET	+	–	NA, S, SXT	wbeO1 + , ace + , zot + , tcpA(ET), rtxC + , ctxA + , rstR(ET), hlyA(ET), Ogawa, ctxB7, intSXT + , intl1–, NA, S, SXT	ND	ND
2021	22	Inaba	+	+	+	ET	+	+	B7	ET	ET	+	–	AMP, NA, S, SXT	wbeO1 + , ace + , zot + , tcpA(ET), rtxC + , ctxA + , rstR(ET), hlyA(ET), Inaba, ctxB7, intSXT + , intl1–, AMP, NA, S, SXT	ND	ND
	1	Ogawa	+	+	+	ET	+	+	B7	ET	ET	+	–	AMP, NA, S, SXT	wbeO1 + , ace + , zot + , tcpA(ET), rtxC + , ctxA + , rstR(ET), hlyA(ET), Ogawa, ctxB7, intSXT + , intl1-, AMP, NA, S, SXT	ND	ND

^*^ET El Tor, *ND* Not Done, *NA* nalidixic acid, *S* streptomycin, *SXT* sulfamethoxazole/trimethoprim, *KF* cephalothin, *CFM* cefixime, *CRO* ceftriaxone, *FEP* cefepime, *MEL* mecillinam, *CIP* ciprofloxacin, *AMP* ampicillin, *ATM* aztreonam

Genomic DNA of all of the isolates (*n* = 172) supported the amplification of *V. cholerae* species-specific gene, *ompW,* and O1-antigen biosynthetic gene, *wbe* (O1), further confirming that the bacteria were *V. cholerae* O1. All *V. cholerae* isolates amplified the CTX-prophage-mediated genes *ctxA*, *ace*, and *zot*, confirming that all of the isolates were toxigenic and harbored CTX-prophage in their genome (Table 1).

All of the *V. cholerae* isolates tested carried the El Tor biotype-specific genes for *tcpA*, *rstR* and *rtxC*. *V. cholerae* strains, classified as hybrid El Tor may carry both *rstR*El Tor and *rstR*Classical in the same genome. However, none of the isolates in this study were found to carry either *rstR*Classical or both *rstR*El Tor and *rstR*Classical together, only *rstR*El Tor was found (Table 1). Gene for cholera toxin B-subunit of classical biotype (*ctxB1*) and Haitian variant type (*ctxB7*) was detected in 87 and 85 *V. cholerae* O1 isolates, respectively (Table 1). PCR results suggested that all the *V. cholerae* O1 strains in this study were atypical El Tor, carrying either *ctxB1* or *ctxB7* and *rstR*El Tor.

**Fig. 2:**
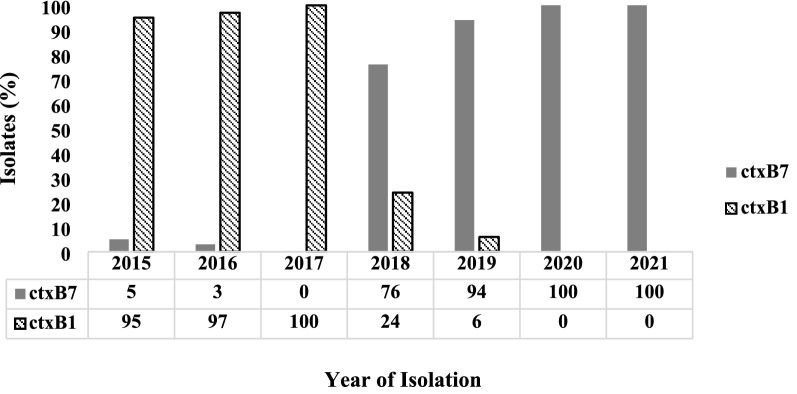
Temporal distribution of *ctxB* alleles among *Vibrio cholerae* O1 strains isolated from Dhaka, 2015–2021

The *ctxB* genotype also showed a temporal shift in predominance during the study period. The classical biotype cholera toxin, *ctxB1*, was predominant having been detected in 95% (n = 19/20), 97% (n = 30/31), and 100% (n = 32/32) of the strains isolated from cholera patients in 2015, 2016 and 2017, respectively (Fig. 2). The Haitian variant cholera toxin, *ctxB7*, was found only in 5% (n = 1/20) and 3% (n = 1/31) *V. cholerae* O1 clinical strains in 2015 and 2016, respectively, while it was not detected in the strains associated with cholera in 2017. Subsequently, *V. cholerae* strains associated with cholera in 2018 and 2019 carried *ctxB7* amongst 76% (n = 16/21) and 94% (n = 10/11) of the strains, but *ctxB1* was detected only in 24% (n = 5/21) and 6% (n = 1/11) of the strains, respectively. All of the *V. cholerae* strains tested in 2020–2021 carried *ctxB7* (n = 23/23, 100%) as the predominant type in Dhaka (Fig. 2).

### Antimicrobial susceptibility patterns

Overall, antimicrobial susceptibility pattern of the studied strains fluctuated slightly across the years. The majority of the strains were multi drug resistant (MDR), showing resistance to at least three different antibiotics (Table 1). Eighteen different antibiotics were tested and 97% (n = 167/172) of the strains circulating among cholera patients in the last 7 years were MDR. All of the isolates were resistant to nalidixic acid (NA), and all, except one strain, sensitive to azithromycin (AZM), erythromycin (E), cephalothin (KF), cefixime (CFM), ceftriaxone (CRO), cefepime (FEP), imipenem (IMP), tetracycline (TE), ciprofloxacin (CIP), levofloxacin (LEV), chloramphenicol (C), gentamicin (CN) and aztreonam (ATM). A unique resistance profile was observed for one of the tested isolates, which was resistant to eleven of the eighteen antibiotics tested, namely ampicillin (AMP), CRO, CIP, mecillinam (MEL), NA, sulfamethoxazole/trimethoprim (SXT), streptomycin (S), KF, CFM, FEP, and ATM. It is important to note that the MIC of the fourth-generation cephalosporin (FEP) for this strain was determined to be ≥ 256 μg/mL. The double disc synergy test (DDST) for ESBL detection was also positive for this strain indicating the production of ESBL.

**Fig. 3:**
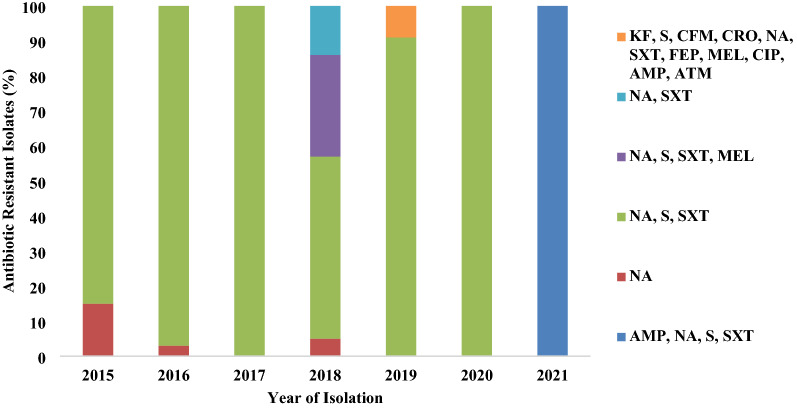
Antibiotic resistance profile of *Vibrio cholerae* O1 strains isolated from Dhaka, 2015–2021. *NA, nalidixic acid; S, streptomycin; SXT, sulfamethoxazole/trimethoprim; KF, cephalothin; CFM, cefixime; CRO, ceftriaxone; FEP, cefepime; MEL, mecillinam; CIP, ciprofloxacin; AMP, ampicillin; ATM, aztreonam

Six distinct resistance profiles were found among the 172 isolates, with a combination of resistance against one to eleven antibiotics during 2015–2021, as shown in Fig. 3. Resistance against S, NA, and SXT was most frequently detected in individual *V. cholerae* isolated from 2015 to 2021 (Fig. 3). Co-occurrence of resistance against S, NA, and SXT has been observed in 84%, 97%, 97%, 54%, 89%, and 97% of the strains isolated from cholera cases during 2015, 2016, 2017, 2018, 2019, and 2020, respectively (Fig. 3). Fluctuation in susceptibility profile was found higher in 2018 when a proportion (29%) of the strains acquired resistance against MEL, which was not recorded in any other year. Contrary to what we observed in 2015–2020, all of the strains associated with cholera were found to have acquired resistance against ampicillin (AMP) in 2021. All of the tested *V. cholerae* strains in 2021 were resistant against AMP, NA, S, and SXT (Fig. 3).

Most of the strains carried *intSXT* (168/172) gene encoding integrase enzyme of the SXT, a mobile genetic element carrying multidrug resistance gene cassettes [10]. Only 3% of the tested strains harbored *intl1* in their genomes, confirming the presence of class 1 integron. None of the tested strains in the present study was found to carry the *intl2* gene encoding class 2 integron (Table 1).

### PCR assays and sequencing of genes responsible for antibiotic resistance

PCR assays for the detection of all of the targeted ESBL genes in the ESBL producing *V. cholerae* O1 isolate (DP19) were negative except *blaPER*-3 (520 bp) and *blaOXA*-1 (564 bp). The strain did not carry any of the genes responsible for quinolone and fluoroquinolone resistance, except for *aac(6’)-Ib-cr* (482 bp) encoding the enzyme acetylase. None of the genes for AmpC group and carbapenemase were detected by PCR assays.

Next, PCR amplicons of the *blaPER*-3, *blaOXA*-1, and *aac(6´)-Ib-cr* genes were sequenced. BLAST homology search revealed that the deduced sequence of 458 bp *blaPER*-3 (Accession No.MK992813) gene had 100% identity with *blaPER*-3 gene of *Aeromonas caviae* reported from China [26]. The sequence of a 496 bp *blaOXA*-1 (Accession No.MK992814) detected in this study had 99.80% identity with *blaOXA*-1 gene found in *Proteus spp*. isolated in China [27]. The DNA sequence of a 415 bp fragment of *aac(6´)-Ib-cr* (Accession No. MK992815) found in *V. cholerae* was identical to that of the *aac(6´)-Ib-cr* existing in *Aeromonas* spp*.* and *Shigella* spp. isolated from water samples [28].

### MLST and MLVA profiles

To determine the genetic relatedness among the isolates we performed molecular fingerprinting analysis on selected strains using Multi Locus Sequence Typing (MLST). We leveraged the genome sequencing data of 30 isolates (20 isolated in 2018, and 10 in 2019) published elsewhere by Monir et al. [23] to deduce their MLST and multi-locus variable number tandem repeat analysis (MLVA) types. MLST typing results based on seven housekeeping genes, *adk, gyrB, mdh, metE, pntA, purM, and pyrC* showed that all of the tested strains belong to sequence type (ST)—69 (Table 1). On the other hand, MLVA results of the tested strains revealed 6 different profiles indicating better discriminatory power of the tool in comparison to MLST for this setting.

## Discussion

*Vibrio cholerae* O1 biotype El Tor, the causative agent of the ongoing 7th cholera pandemic, continues to evolve and cause more severe disease acquiring the classical biotype cholera toxin (*ctxB1* and *ctxB7*), and related classical biotype attributes under the El Tor biotype background [4, 9, 29]. The present research provides insights into the current scenario of cholera in Dhaka, Bangladesh showing the temporal changes in serotype and molecular profiles, including the antimicrobial resistance (AMR) patterns of *V. cholerae* O1 biotype El Tor strains associated with endemic cholera from 2015 to 2021. Our results reaffirm that endemic cholera occurs each year in Bangladesh with *V. cholerae* O1 biotype El Tor strains showing temporal shifts in serotype, *ctxB* genotype, and AMR profile, and call for routine monitoring to aid intervention and preventive measures against the persistent disease resulting in significant morbidity and mortality each year in Bangladesh.

In *V. cholerae*, serotype switching from Inaba to Ogawa, and back to Inaba, occurs in every 2–3 years [11, 12], although the epidemiological significance of the sero-switching is not fully understood for cholera. In the present study, a temporal shift in the predominance of *V. cholerae* serotype was observed twice during 2015–2021, once from Inaba to Ogawa in 2018, and Ogawa to Inaba in 2020. *V. cholerae* associated with endemic cholera in Dhaka had also shown transient switching of *ctxB*1 to *ctxB*7 during 2018–2019, as reported earlier from India, and then in Bangladesh in the subsequent years [9, 10]. It is therefore, evident from the results presented in this study that *V. cholerae* O1 strains carrying *ctxB7*, which is found in association with cholera in Haiti (Haitian variant), was responsible for the recent cholera outbreaks in Dhaka. Our data appear in agreement with reports from other regions of Asia and Africa [30]. Several previous reports and analysis on the origin of Haitian cholera outbreak suggested that *ctxB7* genotype existed in South Asia before 2010. In fact, it was shown that the strains carrying *ctxB7* allele originated in South Asia. For example, there are a few studies which reported that *ctxB7* allele was circulating among *V. cholerae* O1 outbreak causing strains isolated from Kolkata in 2006 and Orissa in 2007 [17]. Epidemiological and phylogenetic analysis particularly supported the hypothesis that strains introduced from Nepal were responsible for the Haitian outbreak in 2010 [7]. Although the biological significance of these mutations in *ctxB* allele is not fully defined, phenotypic analysis revealed that Haitian stains have increased production of cholera toxin and other virulence factors thus contributing to the infectious propensity of *V. cholerae* O1 [31].

When the relatedness of the strains from the transition period (2018–2019) was compared using multi-locus sequence typing (MLST), all isolates belonged to the same MLST type—ST69 (Table 1). This is in agreement with previous report on clinical *V. cholerae* O1 isolated from an urban community in Dhaka, which also belonged to ST69 [32]. Recent studies in Asia and Africa also reported that the majority of the seventh pandemic El Tor (7PET) *V. cholerae* O1 strains belongs to ST69 [33, 34]. It corroborates the observation that the recent epidemic cholera outbreak in Dhaka is predominantly caused by the 7PET lineage, although further study is needed to confirm the clonal relatedness of *V. cholerae* strains responsible for endemic cholera in Bangladesh. Our multi-locus variable-number-tandem-repeat analysis (MLVA) results however differentiated the ST69 strains in the present study into 6 different MLVA types. Although whole genome analysis would be needed to better understand the relatedness, a recent study conducted on *V. cholerae* El Tor isolated from clinical and water sources in Dhaka, Bangladesh, showed similar results with 124 distinct MLVA types from among 621 strains [35].

Temporal switching of serotype and *ctxB* allele is common among *V. cholerae* O1 strains associated with endemic cholera. These strains can usually be divided into two clades at a genomic level—one clade represents strains predominantly having Inaba serotype and carrying *ctxB1* allele while the other clade includes strains of the Ogawa serotype carrying *ctxB7* allele [12, 36]. As a result, switching of serotype and *ctxB* allele usually occurs simultaneously. Similarly, here we noted that during the transition period of serotype conversion and *ctxB* allele switching, the strains had a change in the predominance of serotypes from Inaba to Ogawa in 2018, which continued until 2019, while *ctxB* genotype switching also occurred from *ctxB1* to *ctxB7* during the same period. However, in 2020, Inaba serotype became the predominant serotype again, but *ctxB* allele switching did not occur. As a result, *ctxB7*, instead of *ctxB1*, were found to be the predominant *ctxB* genotype among Inaba strains. These results suggest that the serotype switching is most likely to be an independent event, not causally related to *ctxB* allele switching.

Antibiotic use is a convention for the clinical management of cholera. For this, it is essential to have an up-to-date surveillance data of antibiotic susceptibility patterns of the pathogen, which tend to change over time [5]. *V. cholerae* is a resident flora of wide ranging natural aquatic environments [37, 38], which usually lack antibiotics and related selection pressure essential for the bacterium to steadily carry antibiotic resistance-related plasmids and other mobile genetic elements. As a result, the organism tends to have a fluctuating antibiotic susceptibility pattern. However, owing to the overuse of antibiotics, recent environmental and clinical *V. cholerae* strains appear to be multi-drug resistant acquiring different resistance genes [39]. Such acquisition of resistance and related genes can be both temporary and persisting, as demonstrated by the results presented in this study. For example, tetracycline (TE) was the priority drug widely used for the treatment of cholera, but its clinical use was reduced drastically because of the emergence of TE resistant *V. cholerae* strains in Asia and Africa [5]. The recently isolated *V. cholerae* O1 strains in South Asia, including Bangladesh, were found to be susceptible against TE [40–42]. In the present study all of the *V. cholerae* isolates tested were susceptible towards TE. This could be due to decrease in the use of TE and lack of antibiotic selection pressure [43, 44]. Nonetheless, the increasing susceptibility of *V. cholerae* to TE indicates the effectiveness of this highly cost-effective drug to be in use again for the treatment of cholera. The transient resistance observed against other antibiotics such as mecillinam and ampicillin indicates exposure of *V. cholerae* O1 to these drugs. Consistent with the results from other studies [5, 42], the observed high susceptibility of *V. cholerae* O1 to azithromycin, chloramphenicol, ciprofloxacin, cephalosporins and carbapenems in the present study shows promise for these drugs to be used for treating cholera.

Recent studies have reported compromised drug susceptibility pattern of *V. cholerae* O1 against newer drugs, including those which are not commonly used for the treatment of cholera [41, 42, 45]. For example, recently isolated *V. cholerae* O1 strains were reported to be resistant against extended spectrum beta-lactam antibiotics, such as the fourth generation cephalosporins. Although the underlying molecular mechanism for the drug resistance is yet to be elucidated, it is presumed to be attributed to the activity of either extended spectrum of β-lactamase (ESBL) or AmpC β-lactamase [41, 45]. Beta-Lactams are broad-spectrum drugs used to treat a wide range of infectious diseases and in post-operative infection management [46]. Thus, increasing resistance against beta-lactams and carriage of multiple beta-lactamase genes could not only hinder the clinical management of cholera but also pose a major threat for other infection control measures. In the present study, we report cholera caused by the lone *V. cholerae* O1 strain that was resistant towards eleven different antibiotics, including the fourth generation cephalosporin (FEP), and carrying ESBL encoding genes *blaPER-*3 and *blaOXA-*1 together with fluoroquinolone resistance gene *aac(6´)-Ib-cr*. To our knowledge, this is the first report of the association of an extensively drug resistant (XDR) *V. cholerae* O1 with cholera in Bangladesh. Previously, chromosomal integration of *blaNDM*-1 was reported from the genome of an XDR *V. cholerae* non-O1/O139 strain associated with diarrhea in India [46]. Although *V. cholerae* can develop AMR via single or multiple point mutations in the chromosome, the bacterium can gain resistance genes via horizontal gene transfer (HGT) from other populations sharing niches with them [46]. Owing to the high genetic plasticity of *V. cholerae*, MDR/XDR strains can emanate through the acquisition of extrachromosomal mobile genetic elements such as self-replicating plasmids or integrative mobile genetic elements (MGEs) including SXT integrating conjugative elements (SXT ICEs), class 1 integrons, and transposable genetic elements [43, 46].

In Silico resistome analysis revealed that the XDR isolate (DP19) possessed a bunch of genes related to antimicrobial resistance phenotypes: *aac(6')-Ib-cr5*, *aph(3'')-Ib*, *aph(6)-Id*, *blaOXA-1*, *blaPER-3*, *catB3*, *catB9*, *dfrA1*, *dfrA15*, *mph(A)*, *sul1*, *sul2*, *tetA(D)*, and *varG*. *blaOXA-1* found in the strain showed 99.88% nucleotide sequence identity with the plasmid-borne gene found in several enteric bacteria such as *Escherichia coli*, *Kleibsiella pneumoniae*, *Shigella*, and *Citrobacter*. *blaPER-3* showed 100% nucleotide sequence similarity with the plasmid borne gene found in a *Vibiro parahaemolyticus* isolate (GenBank: KY014464.1). In addition to the *blaOXA-1* and *blaPER-3* beta-lactamase genes, for several other antibiotic resistance genes (*catB3*, *aac(6')-Ib-cr5*, *dfrA1*, *dfrA15*, *mph(A)*, *tetA(D)*, *aph(6)-Id*, *aph(3'')-Ib*, *sul2*, *and sul1)*, identical homologues found in the database are all listed as plasmid borne sourcing in marine and enteric bacteria. These indicates that the resistance related gene cluster in the XDR strain of this study might also be plasmid mediated. However, we could not isolate any plasmid from the strain using plasmid extraction kit as well as following the standard alkaline lysis method. There is a possibility of the presence of a large plasmid and perhaps modification of the protocol would enable isolation of plasmid from the strains, if there is any. In the present study, we also detected SXT-related integrase (*intSXT*) in all of the *V. cholerae* isolates that were resistant to S and SXT, indicating the possible role of SXT/R391 ICE in carrying multiple drug resistance genes for the bacterium.

## Conclusion

The treatment regimen and prevention of cholera relies greatly on deciphering the dynamics of genotypic and phenotypic characteristics, of *V. cholerae*, the causative agent of cholera. In this study, we present data on the genotypic and phenotypic characteristics, including the serotype profiles and drug resistance of *V. cholerae* O1 associated with endemic cholera in Dhaka, Bangladesh, between 2015 and 2021. This study provides new insights on the temporal genetic changes in serotype, genotypes of major virulence genes, and drug resistance profiles of *V. cholerae*, including the emergence of MDR/XDR *V. cholerae* resistant to eleven of eighteen tested drugs including resistance to fourth-generation cephalosporin (cefepime) and aztreonam, which would be important in designing therapeutic intervention and preventive measures for the deadly disease in the global hotspot of transmission. The observed serotype switching occurred among the strains with the Inaba serotype predominant over Ogawa for most of the 7 year study period from 2015 to 2021. Also, we have recorded the transition of the *ctxB* genotype in 2018 from *ctxB1* to *ctxB7*, which became predominant in 2019, and continued as the predominant *ctxB* genotype until 2021. Though it is difficult to foresee how the changing genotype and phenotypic characteristics of *V. cholerae* O1 will affect cholera epidemiology in this region, our results underscore the need for routine monitoring to decide on effective intervention and preventive measures against cholera.

## Data Availability

The PCR amplicon sequence data of blaPER-3, blaOXA-1, and aac(6´)-Ib-cr genes were deposited in NCBI Gene Bank under Accession No. MK992813, MK992814 and MK992815, respectively. All the data are available on request from the corresponding author.
